# Supplementary material for: Predicting Health-Related Quality of Life Using Social Determinants of Health: A Machine Learning Approach with the All of Us Cohort
Source: Bioengineering (Basel). 2025 Feb 9;12(2):166. doi: 10.3390/bioengineering12020166 (PMC11851811; doi:10.3390/bioengineering12020166)
Supplement: Supplementary file 1 [file bioengineering-12-00166-s001.zip › bioengineering-3451424-supplementary.pdf]

List of abbreviations and acronyms

AoU	All of Us
AROC	Area Under the Receiver Operating Characteristic Curve
CKD	Chronic Kidney Disease
DM	Diabetes Mellitus
HF	Heart Failure
HRQOL	Health-Related Quality of Life
ML	Machine learning
RF	RandomForest
SDOH	Social Determinants of Health
XGBoost	Extreme gradient boosting

Table S1: Participant Characteristics

Variables	Total=97,175
Race	
Black or African American	7160 (7.4%)
Whites	78,723 (81%)
others	11292(12.6%)
General health	
Improved	81820(84%)
Unimproved	15355(16%)
HRQOL	
Improved	88417(91%)
Not improved	8758 (9%)
Physical health	
Improved	78981(81.2%)
Not improved	18194 (19.8%)
Mental health	
Improved	84679 (87%)
Not improved	12496 (13%)

DM	
Yes	9,117(9.4%)
No	88058(90.6)
CKD	
Yes	4799 (5%)
No	92376 (95%)
HF	
Yes	3381 (3.5%)
No	93794 (96.5%)
Asthma	
Yes	10219 (10.5%)
No	86956(89.5%)
In the last month, how often have you felt that things were going your way?	
Almost never	7297
Sometimes	26241
Very often	63637
In the last month, how often have you felt that you were on top of things?	
Almost never	8158 (8.3%)
Sometimes	24,200(25%)
Very often	64,817 (66.7%)
How often do you feel God's (or a higher power's) presence	
Never	49951
Rarey	27091
Sometimes	20133
Delayed medication filling to save money	
Yes	12,232
No	84943
Skipp medication to save money	
Yes	6739
No	90436

In the last month, how often have you been upset because of something that happened unexpectedly?	
Sometimes	61471
Never	35704
How often do you feel left out?	
Never	33545
Rarey	36732
Sometimes	26898
The crime rate in my neighborhood makes it unsafe to go on walks during the day	
Strongly disagree	69813
Agree	27362
In the last month, how often have you been able to control irritations in your life?	
Often	33593
Never	3092
Sometimes	32167
Very Often	28323
How often do you feel like a doctor or nurse is not listening to you	
Most of the time	3728
Never	3447
How much you agree or disagree that your neighborhood is safe?	
agree	58425
Strongly agree	38750
Specialist	
Can’t afford	78574
Can afford	18601
How much you agree or disagree that there is too much drug use in your neighborhood?	
Agree	7786

Disagree	37054
Strongly disagree	52335
How often do you fell that you can find companionship when you want it?	
Often	63506
Rarey	6321
Sometimes	27348
How much you agree or disagree that people in your neighborhood generally get along with each other?	
Agree	56258
Strongly agree	24992
Neutral	15925
How often do you go to religious meetings or services?	
I am not religious	57119
Rarely	40,056
In the last month, how often have you been angered because of things that were outside of your control?	
Never	47970
Sometimes	49,205
isolated	
Never	42504
Rarely	31124
Sometimes	23547
Emergency	
Afford	89298
Not afford	7877
Ethnicity	
Not Hispanic Latino	88912
Hispanic Latino	8263

In the last month, how often have you found that you could not cope with things	
Never	65587
Rarely	31588
How often do you feel deep inner peace	
Many times, a day	54404
Once in a while	42771
Sometimes	18855
Very often	78320
Affordability of follow-up visit	
Afford	79826
Not afford	17349
How often are you treated with less courtesy than other people	
A few times a month	5809
Never	39450
A few times a year	26143
Less than once a year	25773

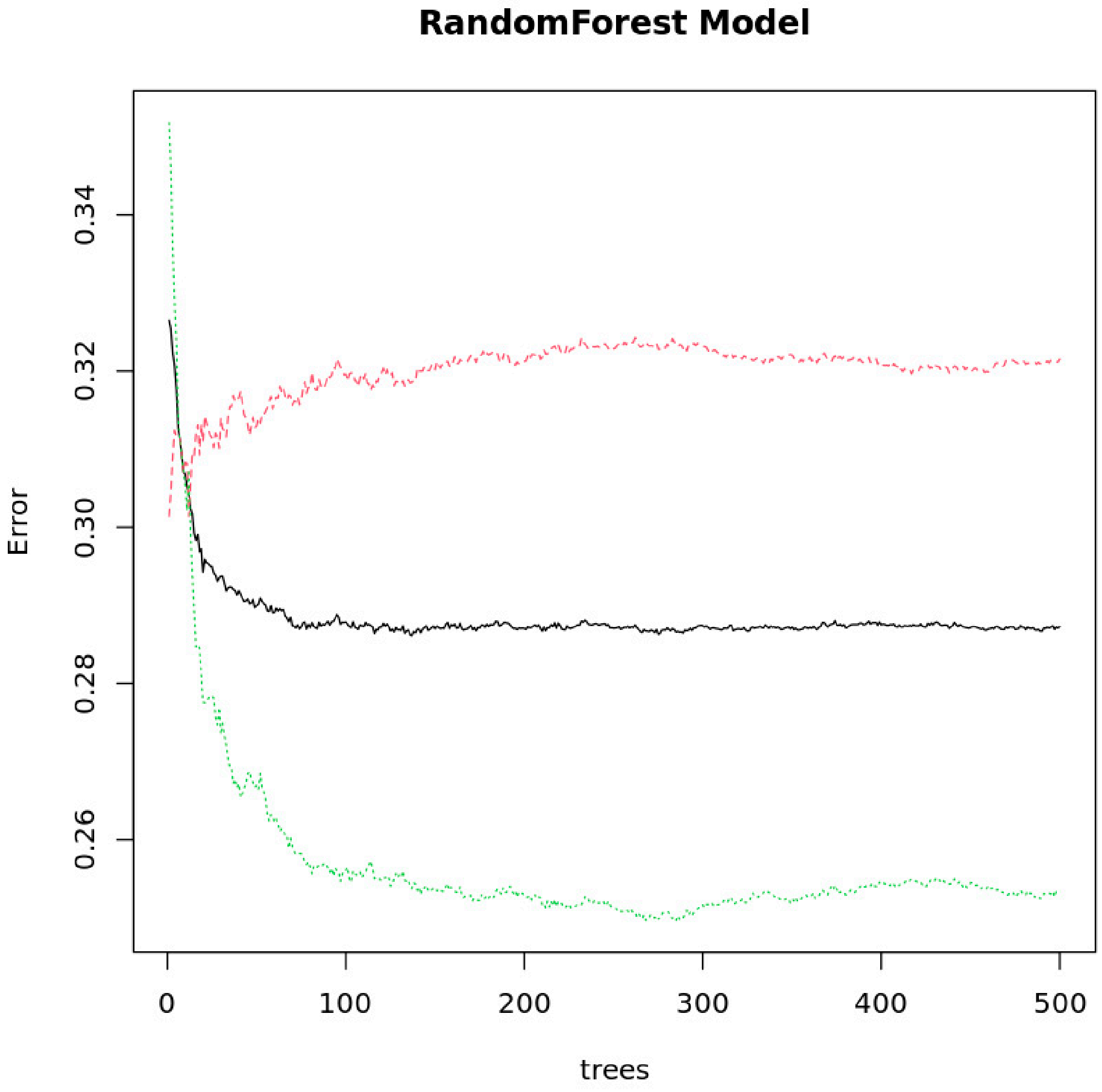

Figure S1: Estimate of the out of bag error (OOB) versus number of trees to optimize the RF model
